# A *metafluid* with multistable density and internal energy states

**DOI:** 10.1038/s41467-022-29048-3

**Published:** 2022-04-05

**Authors:** Ofek Peretz, Ezra Ben Abu, Anna Zigelman, Sefi Givli, Amir D. Gat

**Affiliations:** Faculty of Mechanical Engineering, Technion - Israel Institute of Technology, Haifa, 3200003 Israel

**Keywords:** Mechanical engineering, Fluids, Thermodynamics

## Abstract

Investigating and tailoring the thermodynamic properties of different fluids is crucial to many fields. For example, the efficiency, operation range, and environmental safety of applications in energy and refrigeration cycles are highly affected by the properties of the respective available fluids. Here, we suggest combining gas, liquid and multistable elastic capsules to create an artificial fluid with a multitude of stable states. We study, theoretically and experimentally, the suspension’s internal energy, equilibrium pressure-density relations, and their stability for both adiabatic and isothermal processes. We show that the elastic multistability of the capsules endows the fluid with multistable thermodynamic properties, including the ability of capturing and storing energy at standard atmospheric conditions, not found in naturally available fluids.

## Abstract (web summary)

Investigating and tailoring the thermodynamic properties of different fluids is crucial to many applied fields such as energy and refrigeration cycles. Here, authors use multistable, gas filled, particles suspension to enhance the macro-properties of thermodynamic fluids.

## Introduction

The thermodynamic properties of fluids are crucial to many fields, and specifically to energy and refrigeration cycles^1,2^. These widespread and essential cycles are leading causes for global warming^3–6^, and in particularly, refrigeration was recently listed as the single most polluting technology^3^, due to fluids used within such cycles. Creating a fluid with exceptional properties can thus be of great practical importance, yielding possibilities of advancement. In this work, we study the properties of a suspension composed of a multitude of lubricated multistable capsules (structures capable of transforming between different equilibrium deformation patterns^7,8^) enclosing a compressible gas and immersed within another fluid. The thermodynamic properties of the suspension are determined by an interaction between the external liquid, the encapsulated gas, and the characteristic elastic energy profile of the capsules. By leveraging the elastic multistability of the suspended capsules, we can produce a fluid with multiple stable density points for a given pressure and temperature states as well as unstable regions.

Multistable structures are a special class of mechanical meta-materials. Meta-materials are architected structures made from assemblies of micro-level building blocks, usually arranged in a repeated pattern. While made from standard materials, the geometry of their building blocks can give rise to unique behaviors^9^. For example, meta-materials have been designed to have “negative mass”^10–12^, to manipulate light^13,14^ and stress waves^15–17^, or to provide ultra-high stiffness to weight ratio^18^. Multistable meta-materials feature a multitude of possible equilibrium configurations for a prescribed load. These meta-stable configurations differ in the state of each mechanical unit (building block), which leads to a highly inhomogeneous and non-affine deformation field for the whole body^19^. Hence, by careful design of their building blocks, these multi-stable structures can be “programmed” to undergo morphological changes in response to external stimuli. The ability to manufacture multi-stable meta-materials at all scales has opened exciting possibilities in a range of applications such as vibration isolation, super-elastic behavior, sensing and actuation, soft robotics, deployable structures and more^20–29^.

**Fig. 1:**
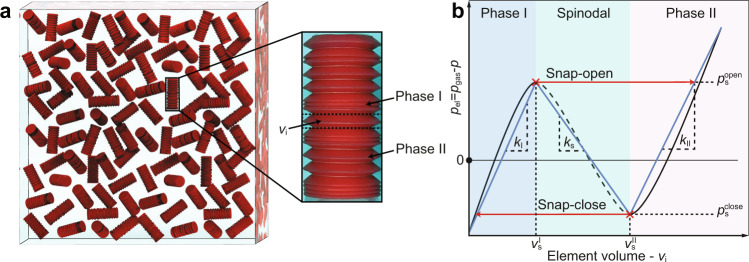
Illustration of a metafluid. **a** Schematic view of a metafluid containing encapsulated gas within multistable capsules, immersed with a second fluid. **b** Pressure (*p*_el_) vs. volume (*v*_i_) relation (shown by black continuous and dashed line) and the tri-linear approximation (shown by blue line) for each individual bi-stable element (*i*) in the multi-stable capsule, where phase I, spinodal, and phase II regions are indicated. The spinodal region defines the region of negative stiffness that separates between phase I and phase II.

In this work, we adopt a similar approach for the creation of multistable metafluids. Metafluids are artificial suspensions that include a mixture of a standard fluid and metamaterial particles engineered to give rise to properties/behaviors that are not found in natural fluids. We study the pressure-density relations, and stability, of a “metafluid", composed of a multitude of multistable gas-filled capsules suspended in a liquid. A simple example of a multistable capsule, used in the current study, is presented in Fig. 1, showing multiple connected bi-stable elements, produced by combining two non-identical conical frusta in opposing orientations. Bi-stability is therefore achieved by switching between extended and collapsed states through inversion of one frustum which creates a significant change in volume. Below, we fully define our model problem, discuss our approach for energy calculation, present our results for stable and unstable equilibria, present experimental data of pressure cycles, examine the use of metafluid in a refrigeration cycle and provide concluding remarks.

## Results

We study the thermodynamic pressure-density relations and their stability for a fluidic suspension composed of multistable elastic capsules, enclosing a compressible gas and immersed in a liquid, as seen in Fig. 1. Each capsule is composed of *n* identical bi-stable elastic elements creating a single encapsulated volume, filled in with a compressible gas. For the sake of simplicity, body forces are hereafter neglected (see detailed analysis in Supplementary Note 4 and Supplementary Movie 3). We examine a control volume which includes a large number of capsules, so that homogenization may be applied, while keeping all physical properties spatially uniform. The density of the external liquid is denoted by *ρ*_l_. The number of capsules per-unit-mass is denoted by Φ. The total mass of the capsules per suspension mass *m* can be calculated via Φ, as *m* = Φ*m*_c_, where *m*_c_ is the mass of a single capsule. We define Ψ as the external fluid mass ratio. Thus, the mass of the external liquid is *m*_l_ = Ψ*m* and the total mass of the capsules is *m*(1 − Ψ). Thus we obtain a Φ-Ψ relation of Φ = (1 − Ψ)/*m*_c_.

Since the external fluid is assumed to be incompressible and thus its volume is constant, the equivalent density *ρ* of the suspension can be expressed by

1 $$\rho ={\left[\frac{1-\Phi {m}_{{{{{{\mathrm{c}}}}}}}}{{\rho }_{{{{{{\mathrm{l}}}}}}}}+\Phi \left({v}_{{{{{{\mathrm{a}}}}}}}+\mathop{\sum }\limits_{i = 1}^{{{{{{\mathrm{n}}}}}}}{v}_{{{{{{\mathrm{i}}}}}}}\right)\right]}^{-1},$$

where *v*_i_ is the gas-filled volume contained within bi-stable element *i*, and *v*_a_ is all the additional gas and solid volume of the capsule.

The volume *v*_i_ is a multi-valued function of the difference between the pressure of the internal gas, denoted as *p*_gas_ and the pressure in the external fluid, denoted simply as *p*. We thus define this pressure difference by *p*_el_ = *p*_gas_ − *p*. We approximate *v*_i_ by the tri-linear model (see Fig. 1b) which is a common and useful approximation that enables a simplified analytic treatment of bi-stability. More specifically, according to this model, when considering the energy relation to the pressure *p*_el_ and element volume *v*_i_, the energy function may be approximated by three quadratic functionals^19,30–34^ leading to piece-wise linear pressure-volume relation.

The tri-linear curve is defined by the stiffnesses *k*_I_ and *k*_II_, and the stability threshold points $({v}_{{{{{{\mathrm{s}}}}}}}^{{{{{{{\mathrm{I}}}}}}}},{p}_{{{{{{\mathrm{s}}}}}}}^{{{{{{{\mathrm{open}}}}}}}})$ and $({v}_{{{{{{\mathrm{s}}}}}}}^{{{{{{{\mathrm{II}}}}}}}},{p}_{{{{{{\mathrm{s}}}}}}}^{{{{{{{\mathrm{close}}}}}}}})$ in phases I and II, respectively (see definitions in Fig. 1b). Thus, the possible pressure range for bi-stable elements in phase I is ${p}_{{{{{{\mathrm{el}}}}}}} \, < \, {p}_{{{{{{\mathrm{s}}}}}}}^{{{{{{{\mathrm{open}}}}}}}}$, in the spinodal region ${p}_{{{{{{\mathrm{s}}}}}}}^{{{{{{{\mathrm{close}}}}}}}} \, < \, {p}_{{{{{{\mathrm{el}}}}}}} \, < \, {p}_{{{{{{\mathrm{s}}}}}}}^{{{{{{{\mathrm{open}}}}}}}}$, and in phase II ${p}_{{{{{{\mathrm{el}}}}}}} \, > \, {p}_{{{{{{\mathrm{s}}}}}}}^{{{{{{{\mathrm{close}}}}}}}}$. These coefficients are commonly evaluated by fitting to an experiment^24,35,36^. Thus, the pressure-volume function for each of the bi-stable elements is given by,

2 $${p}_{{{{{{\mathrm{el}}}}}}}=\left\{\begin{array}{c}{p}_{{{{{{\mathrm{el}}}}}}}^{{{{{{{\mathrm{I}}}}}}}}\\ {p}_{{{{{{\mathrm{el}}}}}}}^{{{{{{\mathrm{s}}}}}}}\\ {p}_{{{{{{\mathrm{el}}}}}}}^{{{{{{{\mathrm{II}}}}}}}}\end{array}\right\}=\left\{\begin{array}{c}{k}_{{{{{{{\mathrm{I}}}}}}}}\left({v}_{{{{{{\mathrm{i}}}}}}}-{v}_{{{{{{\mathrm{s}}}}}}}^{{{{{{{\mathrm{I}}}}}}}}\right)+{p}_{{{{{{\mathrm{s}}}}}}}^{{{{{{{\mathrm{open}}}}}}}}\\ {k}_{{{{{{\mathrm{s}}}}}}}\left({v}_{{{{{{\mathrm{i}}}}}}}-{v}_{{{{{{\mathrm{s}}}}}}}^{{{{{{{\mathrm{I}}}}}}}}\right)+{p}_{{{{{{\mathrm{s}}}}}}}^{{{{{{{\mathrm{open}}}}}}}}\\ {k}_{{{{{{{\mathrm{II}}}}}}}}\left({v}_{{{{{{\mathrm{i}}}}}}}-{v}_{{{{{{\mathrm{s}}}}}}}^{{{{{{{\mathrm{II}}}}}}}}\right)+{p}_{{{{{{\mathrm{s}}}}}}}^{{{{{{{\mathrm{close}}}}}}}}\end{array}\right\},\quad \begin{array}{c}{v}_{{{{{{\mathrm{i}}}}}}} \, < \, {v}_{{{{{{\mathrm{s}}}}}}}^{{{{{{{\mathrm{I}}}}}}}}\\ {v}_{{{{{{\mathrm{s}}}}}}}^{{{{{{{\mathrm{I}}}}}}}} \, < \, {v}_{{{{{{\mathrm{i}}}}}}} \, < \, {v}_{{{{{{\mathrm{s}}}}}}}^{{{{{{{\mathrm{II}}}}}}}}\\ {v}_{{{{{{\mathrm{s}}}}}}}^{{{{{{{\mathrm{II}}}}}}}} \, < \, {v}_{{{{{{\mathrm{i}}}}}}}\end{array},$$

where *k*_s_ is determined by requiring continuity between the spinodal region and phases I and II, yielding ${k}_{{{{{{\mathrm{s}}}}}}}=({p}_{{{{{{\mathrm{s}}}}}}}^{{{{{{{\mathrm{close}}}}}}}}-{p}_{{{{{{\mathrm{s}}}}}}}^{{{{{{{\mathrm{open}}}}}}}})/({v}_{{{{{{\mathrm{s}}}}}}}^{{{{{{{\mathrm{II}}}}}}}}-{v}_{{{{{{\mathrm{s}}}}}}}^{{{{{{{\mathrm{I}}}}}}}}) \, < \, 0$.

Next, we address the total gas-filled volume of a capsule, which is composed of the sum of all volumes of the *n* bi-stable elements (given by $\mathop{\sum }\nolimits_{i = 1}^{{{{{{\mathrm{n}}}}}}}{v}_{{{{{{\mathrm{i}}}}}}}$ and additional gas-filled volume *v*_a,gas_). Note that the pressure acting on all elements is identical and depends only on the sum of all volumes, so that the overall pressure-volume relation of the capsule is dictated by the number of elements in phase I (*n*_I_), in phase II (*n*_II_), and in the spinodal (*n*_s_). For convenience we denote the vector containing the number of elements in each multistable state as the permutation,

3 $$\mathop{{{{{{{{\bf{per}}}}}}}}}\limits^{-\!\longrightarrow}=\{{n}_{{{{{{{\mathrm{I}}}}}}}},{n}_{{{{{{\mathrm{s}}}}}}},{n}_{{{{{{{\mathrm{II}}}}}}}}\}.$$

In other words, different permutations lead to different pressure-volume relations which are independent of the specific arrangement within a permutation. Thus, a capsule with *n* bi-stable elements has (*n*^2^ + 3*n* + 2)/2 possible permutations. In the experiment, each capsule contains *n* = 18 elements which yields 190 possible permutations. Considering that the pressure acting on all elements is identical and equal to *p*_el_, we can calculate the volume of an element in each phase according to Eq. (2). Denoting the volume in each phase by *v*_I_(*p*_el_), *v*_s_(*p*_el_), *v*_II_(*p*_el_), we obtain the total volume of gas within a capsule as $\mathop{{{{{{{{\bf{per}}}}}}}}}\limits^{-\!\longrightarrow}\cdot \{{v}_{{{{{{{\mathrm{I}}}}}}}}({p}_{{{{{{\mathrm{el}}}}}}}),{v}_{{{{{{{\mathrm{s}}}}}}}}({p}_{{{{{{\mathrm{el}}}}}}}),{v}_{{{{{{{\mathrm{II}}}}}}}}({p}_{{{{{{\mathrm{el}}}}}}})\}+{v}_{{{{{{\mathrm{a}}}}}},{{{{{\mathrm{gas}}}}}}}$.

### Internal energy

We now calculate the internal energy for all of the possible permutations {*n*_I_, *n*_s_, *n*_II_} of the suspension. For the current analysis, we neglect the compressibility of the external fluid and assume that all capsules are identical. Thus, the internal energy of the suspension is composed of the total energy of the ideal gas, denoted by *U*_gas_, and the energy of the multistable elastic capsules, denoted by *U*_el_.

The elastic energy *U*_el_ of a bi-stable element *i* for the three possible phases is given by integration of (2) over *v*_i_, yielding

4 $$\left\{\begin{array}{c}{U}_{{{{{{\mathrm{el}}}}}}}^{{{{{{{\mathrm{I}}}}}}}}\\ {U}_{{{{{{\mathrm{el}}}}}}}^{s}\\ {U}_{{{{{{\mathrm{el}}}}}}}^{{{{{{{\mathrm{II}}}}}}}}\end{array}\right\}=\left\{\begin{array}{c}{k}_{{{{{{{\mathrm{I}}}}}}}}{\left({v}_{{{{{{\mathrm{i}}}}}}}-{v}_{{{{{{\mathrm{s}}}}}}}^{{{{{{{\mathrm{I}}}}}}}}\right)}^{2}/2+{p}_{{{{{{\mathrm{s}}}}}}}^{{{{{{{\mathrm{open}}}}}}}}{v}_{{{{{{\mathrm{i}}}}}}}\\ {k}_{{{{{{\mathrm{s}}}}}}}{\left({v}_{{{{{{\mathrm{i}}}}}}}-{v}_{{{{{{\mathrm{s}}}}}}}^{{{{{{{\mathrm{I}}}}}}}}\right)}^{2}/2+{p}_{{{{{{\mathrm{s}}}}}}}^{{{{{{{\mathrm{open}}}}}}}}{v}_{{{{{{\mathrm{i}}}}}}}\\ {k}_{{{{{{{\mathrm{II}}}}}}}}{\left({v}_{{{{{{\mathrm{i}}}}}}}-{v}_{{{{{{\mathrm{s}}}}}}}^{{{{{{{\mathrm{II}}}}}}}}\right)}^{2}/2+{p}_{{{{{{\mathrm{s}}}}}}}^{{{{{{{\mathrm{close}}}}}}}}{v}_{{{{{{\mathrm{i}}}}}}}\end{array}\right\},\quad \begin{array}{c}{v}_{{{{{{\mathrm{i}}}}}}} \, < \, {v}_{{{{{{\mathrm{s}}}}}}}^{{{{{{{\mathrm{I}}}}}}}}\\ {v}_{{{{{{\mathrm{s}}}}}}}^{{{{{{{\mathrm{I}}}}}}}} \, < \, {v}_{{{{{{\mathrm{i}}}}}}} \, < \, {v}_{{{{{{\mathrm{s}}}}}}}^{{{{{{{\mathrm{II}}}}}}}}\\ {v}_{{{{{{\mathrm{s}}}}}}}^{{{{{{{\mathrm{II}}}}}}}} \, < \, {v}_{{{{{{\mathrm{i}}}}}}}\\ \end{array}$$

and the total elastic potential energy per-unit-mass is thus

5 $${U}_{{{{{{\mathrm{el}}}}}}}=\Phi \cdot \mathop{{{{{{{{\bf{per}}}}}}}}}\limits^{-\!\longrightarrow}\cdot \{{U}_{{{{{{\mathrm{el}}}}}}}^{{{{{{{\mathrm{I}}}}}}}},{U}_{{{{{{\mathrm{el}}}}}}}^{{{{{{\mathrm{s}}}}}}},{U}_{{{{{{\mathrm{el}}}}}}}^{{{{{{{\mathrm{II}}}}}}}}\}.$$

To calculate *U*_gas_ we assume an ideal gas model, *p*_gas_ = *ρ*_gas_*RT*. For isothermal processes, we thus obtain the relation ${p}_{{{{{{\mathrm{gas}}}}}}}={m}_{{{{{{\mathrm{g}}}}}}}RT/({v}_{{{{{{\mathrm{a,gas}}}}}}}+\mathop{{{{{{{{\bf{per}}}}}}}}}\limits^{-\!\longrightarrow}\cdot \{{v}_{{{{{{{\mathrm{I}}}}}}}},{v}_{{{{{{{\mathrm{s}}}}}}}},{v}_{{{{{{{\mathrm{II}}}}}}}}\})$, where *R* is the specific gas constant, *T* is the constant temperature, *m*_g_ is the mass of the gas within the capsule. For adiabatic processes, assuming ideal gas and isentropic compression/expansion, the gas pressure is given by ${p}_{{{{{{\mathrm{gas}}}}}}}/{p}_{{{{{{\mathrm{atm}}}}}}}={[{v}_{{{{{{\mathrm{atm}}}}}}}/({v}_{{{{{{\mathrm{a}}}}}},{{{{{\mathrm{gas}}}}}}}+\mathop{{{{{{{{\bf{per}}}}}}}}}\limits^{-\!\longrightarrow}\cdot \{{v}_{{{{{{{\mathrm{I}}}}}}}},{v}_{{{{{{{\mathrm{s}}}}}}}},{v}_{{{{{{{\mathrm{II}}}}}}}}\})]}^{\gamma }$, where *p*_atm_ is atmospheric pressure, and *v*_atm_ is the volume taken by the gas contained within a single capsule at atmospheric pressure. In the adiabatic case, the temperature of the gas within the capsules will change during pressure variations according to $T={T}_{0}{(p/{p}_{0})}^{1-1/\gamma }$, but not the temperature of the external incompressible fluid. These spatial temperature variations may yield heat transfer between the gas, solid and liquid, and more complex dynamics. To allow simple treatment of this case, we assume in the current analysis that an adiabatic process corresponds to the more strict requirement of no heat transfer from the encapsulated gas.

The gas energy per-unit-mass of the suspension is defined as

6 $${U}_{{{{{{\mathrm{gas}}}}}}}=-\Phi \int\nolimits_{{v}_{{{{{{\mathrm{atm}}}}}}}}^{{v}_{{{{{{\mathrm{a}}}}}},{{{{{\mathrm{gas}}}}}}}+\mathop{{{{{{{{\bf{per}}}}}}}}}\limits^{-\!\longrightarrow}\cdot \{{v}_{{{{{{{\mathrm{I}}}}}}}},{v}_{{{{{{{\mathrm{s}}}}}}}},{v}_{{{{{{{\mathrm{II}}}}}}}}\}}{p}_{{{{{{\mathrm{gas}}}}}}}{{{{{\mathrm{d}}}}}}v,$$

and thus the total energy of the system for an adiabatic process is given by *U*_tot_ = *U*_gas_ + *U*_el_ + *U*_ex_,

7 $${U}_{{{{{{\mathrm{tot}}}}}}}=	\frac{\Phi {p}_{{{{{{\mathrm{atm}}}}}}}{v}_{{{{{{\mathrm{atm}}}}}}}}{\gamma -1}\left[{\left(\frac{{v}_{{{{{{\mathrm{atm}}}}}}}}{{v}_{{{{{{\mathrm{a}}}}}},{{{{{\mathrm{gas}}}}}}}+\mathop{{{{{{{{\bf{per}}}}}}}}}\limits^{-\!\longrightarrow}\cdot \{{v}_{{{{{{{\mathrm{I}}}}}}}},{v}_{{{{{{{\mathrm{s}}}}}}}},{v}_{{{{{{{\mathrm{II}}}}}}}}\}}\right)}^{\gamma -1}-1\right]\\ 	+\Phi \left\{\begin{array}{c}{k}_{{{{{{{\mathrm{I}}}}}}}}{\left({v}_{{{{{{{\mathrm{I}}}}}}}}-{v}_{{{{{{\mathrm{s}}}}}}}^{{{{{{{\mathrm{I}}}}}}}}\right)}^{2}/2+{p}_{{{{{{\mathrm{s}}}}}}}^{{{{{{{\mathrm{open}}}}}}}}{v}_{{{{{{{\mathrm{I}}}}}}}}\\ {k}_{{{{{{\mathrm{s}}}}}}}{\left({v}_{{{{{{{\mathrm{s}}}}}}}}-{v}_{{{{{{\mathrm{s}}}}}}}^{{{{{{{\mathrm{I}}}}}}}}\right)}^{2}/2+{p}_{{{{{{\mathrm{s}}}}}}}^{{{{{{{\mathrm{open}}}}}}}}{v}_{{{{{{{\mathrm{s}}}}}}}}\\ {k}_{{{{{{{\mathrm{II}}}}}}}}{\left({v}_{{{{{{{\mathrm{II}}}}}}}}-{v}_{{{{{{\mathrm{s}}}}}}}^{{{{{{{\mathrm{II}}}}}}}}\right)}^{2}/2+{p}_{{{{{{\mathrm{s}}}}}}}^{{{{{{{\mathrm{close}}}}}}}}{v}_{{{{{{{\mathrm{II}}}}}}}}\end{array}\right\}\cdot \mathop{{{{{{{{\bf{per}}}}}}}}}\limits^{-\!\longrightarrow}\\ 	+\Phi p\left[{v}_{{{{{{\mathrm{a}}}}}},{{{{{\mathrm{gas}}}}}}}+\mathop{{{{{{{{\bf{per}}}}}}}}}\limits^{-\!\longrightarrow}\cdot \{{v}_{{{{{{{\mathrm{I}}}}}}}},{v}_{{{{{{{\mathrm{s}}}}}}}},{v}_{{{{{{{\mathrm{II}}}}}}}}\}-{v}_{{{{{{\mathrm{atm}}}}}}}\right]$$

where the last RHS term is the external work energy *U*_ex_ and *p* is the dictated pressure in the external fluid. For an isothermal process the first RHS term is replaced with $\Phi {p}_{{{{{{\mathrm{atm}}}}}}}\ln \big[({v}_{{{{{{\mathrm{a}}}}}},{{{{{\mathrm{gas}}}}}}}+\mathop{{{{{{{{\bf{per}}}}}}}}}\limits^{-\!\longrightarrow}\cdot \{{v}_{{{{{{{\mathrm{I}}}}}}}},{v}_{{{{{{{\mathrm{s}}}}}}}},{v}_{{{{{{{\mathrm{II}}}}}}}}\})/{v}_{{{{{{\mathrm{atm}}}}}}}\big]$.

Equilibrium of the system occurs when the elastic force in all bi-stable elements is balanced by the difference between the external pressure, and the pressure of the internal gas. In the current analysis, all bi-stable elements are identical, and can be in one of three possible states (phase I, spinodal, and phase II). Thus, the equilibrium values in each of the three possible states can be calculated from the following relations,

8 $${k}_{{{{{{{\mathrm{I}}}}}}}}\left({v}_{{{{{{{\mathrm{I}}}}}}}}-{v}_{{{{{{\mathrm{s}}}}}}}^{{{{{{{\mathrm{I}}}}}}}}\right)+{p}_{{{{{{\mathrm{s}}}}}}}^{{{{{{{\mathrm{open}}}}}}}} 	={k}_{{{{{{\mathrm{s}}}}}}}\left({v}_{{{{{{{\mathrm{s}}}}}}}}-{v}_{{{{{{\mathrm{s}}}}}}}^{{{{{{{\mathrm{I}}}}}}}}\right)+{p}_{{{{{{\mathrm{s}}}}}}}^{{{{{{{\mathrm{open}}}}}}}}={k}_{{{{{{{\mathrm{II}}}}}}}}\left({v}_{{{{{{{\mathrm{II}}}}}}}}-{v}_{{{{{{\mathrm{s}}}}}}}^{{{{{{{\mathrm{II}}}}}}}}\right)+{p}_{{{{{{\mathrm{s}}}}}}}^{{{{{{{\mathrm{close}}}}}}}}\\ 	={p}_{{{{{{\mathrm{atm}}}}}}}{\left[\frac{{v}_{{{{{{\mathrm{atm}}}}}}}}{{v}_{{{{{{\mathrm{a}}}}}},{{{{{\mathrm{gas}}}}}}}+\mathop{{{{{{{{\bf{per}}}}}}}}}\limits^{-\!\longrightarrow}\cdot \{{v}_{{{{{{{\mathrm{I}}}}}}}},{v}_{{{{{{{\mathrm{s}}}}}}}},{v}_{{{{{{{\mathrm{II}}}}}}}}\}}\right]}^{\gamma }-p.$$

Equation (8) describes an adiabatic process, and for an isothermal process the first RHS term is replaced with $({m}_{{{{{{\mathrm{g}}}}}}}RT)/({v}_{{{{{{\mathrm{a}}}}}},{{{{{\mathrm{gas}}}}}}}+\mathop{{{{{{{{\bf{per}}}}}}}}}\limits^{-\!\longrightarrow}\cdot \{{v}_{{{{{{{\mathrm{I}}}}}}}},{v}_{{{{{{{\mathrm{s}}}}}}}},{v}_{{{{{{{\mathrm{II}}}}}}}}\})$.

**Fig. 2:**
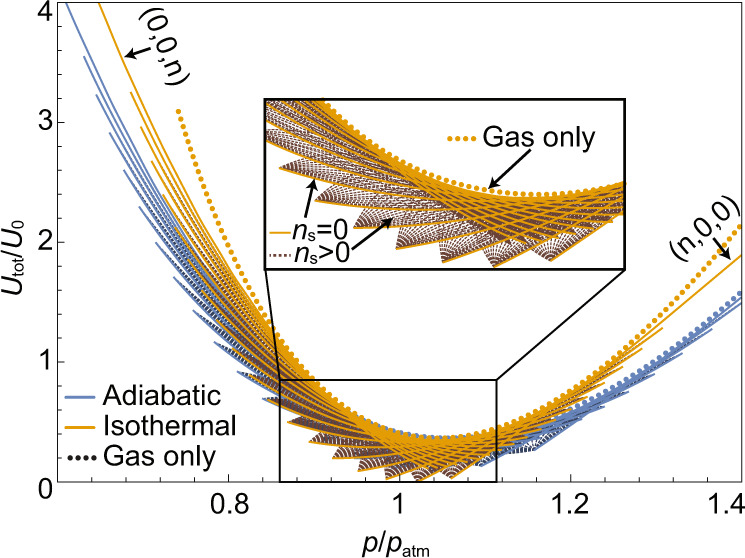
Multiple possible internal energy states of a metafluid. The figure presents analytical results for *U*_tot_/*U*_0_, vs. pressure, *p*/*p*_atm_, where *U*_0_ = Φ*p*_atm_*v*_atm_. All system parameters were taken from experimental measurements and are defined below (see caption of Fig. 4). Adiabatic and isothermal cases are shown by blue and orange lines, respectively, where dashed lines denote states with bi-stable elements in the spinodal (*n*_s_ ≠ 0). Dotted curves of the corresponding colors represent the energy-pressure relation for an ideal gas. The multiple possible internal energies per external pressure allow to store, or release, energy at atmospheric conditions. The density of possible states decreases as the distance to the global minimum increases.

Solving Eq. (8) for the equilibrium values *v*_I_, *v*_s_ and *v*_II_, and substituting these into the energy equation (7) allows to calculate the energy of the suspension per given permutation $\mathop{{{{{{{{\bf{per}}}}}}}}}\limits^{-\!\longrightarrow}=\{{n}_{{{{{{{\mathrm{I}}}}}}}},{n}_{{{{{{\mathrm{s}}}}}}},{n}_{{{{{{{\mathrm{II}}}}}}}}\}$ and external pressure *p*. Each permutation is only valid in a specific range of external pressures, as defined in Eq. (4). In Fig. 2 we present all of the available energy equilibria states, for multistable capsules composed of 18 bi-stable elements, with 190 possible permutations. Both isothermal (orange lines) and adiabatic (blue lines) processes are presented. The lines are determined by changing the external pressure, for different permutations of the phases of the bi-stable elements. The results show that the multi-stability extends the possible state of an ideal gas from a single line to a region containing multitude of possible energies. Different permutations provide different energy-pressure curves, corresponding to a fluid with different properties. Changing the properties of the fluid can be achieved by increasing/reducing the pressure beyond the range of a specific permutation. In addition to changing fluid properties, such transitions between different equilibrium states allow the fluid to store and release energy, as discussed below.

### Stability and the equation of state

We examine the stability of equilibrium states by considering the Hessian of the total energy *U*_tot_, given by *H*_ij_ = ∂^2^*U*_tot_/∂*v*_i_∂*v*_j_. An equilibrium configuration is stable if it corresponds to a local minimum of the energy, requiring positive definite Hessian. In order to obtain the Hessian in terms of all *n* degrees of freedom, *v*_i_, we note that $\mathop{{{{{{{{\bf{per}}}}}}}}}\limits^{-\!\longrightarrow}\cdot \{{v}_{{{{{{{\mathrm{I}}}}}}}},{v}_{{{{{{\mathrm{s}}}}}}},{v}_{{{{{{{\mathrm{II}}}}}}}}\}=\mathop{\sum }\nolimits_{j = 1}^{{{{{{\mathrm{n}}}}}}}{v}_{{{{{{\mathrm{j}}}}}}}$. Substituting this relation into Eq. (7) and differentiating the total energy with respect to the degrees of freedom, *v*_i_, yields the following set of *n* equations

9 $$\frac{\partial {U}_{{{{{{\mathrm{tot}}}}}}}}{\partial {v}_{{{{{{\mathrm{i}}}}}}}}={p}_{{{{{{\mathrm{el}}}}}}}^{\alpha (i)}\left({v}_{{{{{{\mathrm{i}}}}}}}\right)+\left(\frac{{v}_{{{{{{\mathrm{atm}}}}}}}}{{v}_{{{{{{\mathrm{a}}}}}},{{{{{\mathrm{gas}}}}}}}+\mathop{\sum }\limits_{j=1}^{{{{{{\mathrm{n}}}}}}}{v}_{{{{{{\mathrm{j}}}}}}}}\right)-p=0,\quad i=1,\ldots ,n,$$

where *α*(*i*) denotes the phase of bi-stable element *i*, *α*(*i*) ∈ {I, *s*, II}. The set of *n* equations in (9) is differentiated again in order to obtain the Hessian matrix,

10 $$\frac{{\partial }^{2}{U}_{{{{{{\mathrm{tot}}}}}}}}{\partial {v}_{{{{{{\mathrm{i}}}}}}}\partial {v}_{{{{{{\mathrm{j}}}}}}}}={\delta }_{{{{{{\mathrm{ij}}}}}}}{k}_{\alpha (i)}+{k}_{{{{{{\mathrm{g}}}}}}},\quad i,j=1,\ldots ,n,$$

where *k*_*α*(*i*)_ (*i* = 1, 2, …,*n*) is the stiffness of the *i-*th bi-stable element and *k*_g_ is the (instantaneous) stiffness of the gas at the equilibrium configuration. For an adiabatic process ${k}_{g}=(\gamma {p}_{{{{{{\mathrm{atm}}}}}}}/{v}_{{{{{{\mathrm{atm}}}}}}}){[{v}_{{{{{{\mathrm{atm}}}}}}}/({v}_{{{{{{\mathrm{a}}}}}},{{{{{\mathrm{gas}}}}}}}+\mathop{{{{{{{{\bf{per}}}}}}}}}\limits^{-\!\longrightarrow}\cdot \{{v}_{{{{{{{\mathrm{I}}}}}}}},{v}_{{{{{{{\mathrm{s}}}}}}}},{v}_{{{{{{{\mathrm{II}}}}}}}}\})]}^{\gamma +1}$ and for isothermal process ${k}_{{{{{{\mathrm{g}}}}}}}={v}_{{{{{{\mathrm{atm}}}}}}}{p}_{{{{{{\mathrm{atm}}}}}}}/{({v}_{{{{{{\mathrm{a}}}}}},{{{{{\mathrm{gas}}}}}}}+\mathop{{{{{{{{\bf{per}}}}}}}}}\limits^{-\!\longrightarrow}\cdot \{{v}_{{{{{{{\mathrm{I}}}}}}}},{v}_{{{{{{{\mathrm{s}}}}}}}},{v}_{{{{{{{\mathrm{II}}}}}}}}\})}^{2}$.

An equilibrium state is stable if and only if the Hessian is positive definite, which occurs if and only if all leading principal minors *A*_j_ (the determinant of the submatrix of first *j* rows and *j* columns) are positive. The principal minors of the current configuration in (10) are

11 $${A}_{j}=\left(\mathop{\prod }\limits_{i = 1}^{j}{k}_{\alpha (i)}\right)\left[1+{k}_{{{{{{\mathrm{g}}}}}}}\mathop{\sum }\limits_{i=1}^{{{{{{\mathrm{j}}}}}}}\frac{1}{{k}_{\alpha (i)}}\right].$$

In order to obtain conditions for positive *A*_j_, we examine separately three possible permutation states. In the first state the permutation is of the form (*n*_I_, 0, *n*_II_) and thus there are no elements in the spinodal region. In this case, since all *k*_*α*(*i*)_ and *k*_g_ are positive, all minors *A*_j_ in (11) are necessarily positive for all *p*. Another possible permutation state is the case of two or more elements in the spinodal region. The symmetry in terms of the degrees of freedom allows us to arbitrarily rearrange the degrees of freedom. We re-order the Hessian so that the first two elements are in the spinodal region, and thus *k*_*α*(1)_ = *k*_*α*(2)_ = *k*_s_ < 0. The first minor in this case is given by *A*_1_ = *k*_g_*k*_s_(1/*k*_g_ + 1/*k*_s_) and is positive only if *k*_g_ > ∣*k*_s_∣. The second minor ${A}_{2}={k}_{{{{{{\mathrm{g}}}}}}}{({k}_{{{{{{\mathrm{s}}}}}}})}^{2}(1/{k}_{{{{{{\mathrm{g}}}}}}}+2/{k}_{{{{{{\mathrm{s}}}}}}})$) is positive only if *k*_g_ < ∣*k*_s_∣/2. Thus, since the two conditions cannot hold simultaneously, if there are at least two elements in the spinodal region, the configuration is unstable for all *k*_g_. Finally, we examine the case of permutations of the form (*n*_I_, 1, *n*_II_), meaning that only a single element is in the spinodal region. We re-order the Hessian so that the *n-*th element is in the spinodal region. Thus all minors *A*_j_, *j* ∈ {1, …, *n* − 1}, are positive. The requirement that the minor *A*_n_ is positive, which is needed for the stability condition to be satisfied, may be expressed for an adiabatic process as

12 $$\frac{{v}_{{{{{{\mathrm{atm}}}}}}}}{\gamma {p}_{{{{{{\mathrm{atm}}}}}}}}{\left(\frac{{v}_{{{{{{\mathrm{a}}}}}},{{{{{\mathrm{gas}}}}}}}+\mathop{{{{{{{{\bf{per}}}}}}}}}\limits^{-\!\longrightarrow}\cdot \{{v}_{{{{{{{\mathrm{I}}}}}}}},{v}_{{{{{{{\mathrm{s}}}}}}}},{v}_{{{{{{{\mathrm{II}}}}}}}}\}}{{v}_{atm}}\right)}^{\gamma +1}+\mathop{{{{{{{{\bf{per}}}}}}}}}\limits^{-\!\longrightarrow}\cdot \left\{\frac{1}{{k}_{{{{{{{\mathrm{I}}}}}}}}},\frac{1}{{k}_{{{{{{{\mathrm{s}}}}}}}}},\frac{1}{{k}_{{{{{{{\mathrm{II}}}}}}}}}\right\} \, < \, 0$$

where {*v*_I_, *v*_s_, *v*_II_} are functions of the external pressure *p*, and are calculated from Eq. (8). For isothermal process the first LHS term is replaced with ${\left({v}_{{{{{{\mathrm{a}}}}}},{{{{{\mathrm{gas}}}}}}}+\mathop{{{{{{{{\bf{per}}}}}}}}}\limits^{-\!\longrightarrow}\cdot \{{v}_{{{{{{{\mathrm{I}}}}}}}},{v}_{{{{{{{\mathrm{s}}}}}}}},{v}_{{{{{{{\mathrm{II}}}}}}}}\}\right)}^{2}/({v}_{{{{{{\mathrm{atm}}}}}}}{p}_{{{{{{\mathrm{atm}}}}}}})$. Obtaining the stability condition allows us to present all possible density-pressure relations which correspond to all permutations of the form $\mathop{{{{{{{{\bf{per}}}}}}}}}\limits^{-\!\longrightarrow}=({n}_{{{{{{{\mathrm{I}}}}}}}},0,{n}_{{{{{{{\mathrm{II}}}}}}}})$ or permutations of the form $\mathop{{{{{{{{\bf{per}}}}}}}}}\limits^{-\!\longrightarrow}=({n}_{{{{{{{\mathrm{I}}}}}}}},1,{n}_{{{{{{{\mathrm{II}}}}}}}})$ along with the condition (12).

**Fig. 3:**
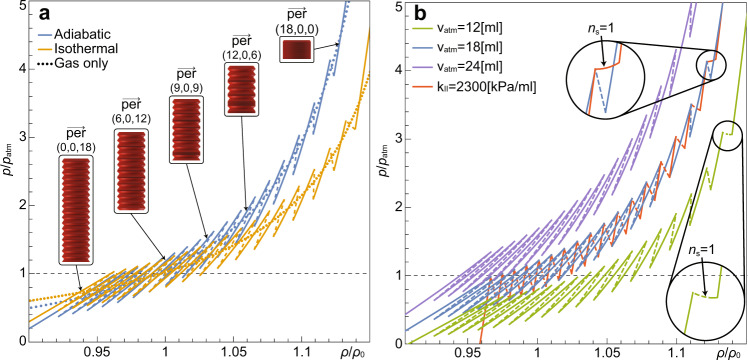
Stability and the equation of state. Analytical results for pressure (*p*/*p*_atm_) vs. density (*ρ*/*ρ*_0_) for stable (solid lines) and unstable (dashed lines) equilibria states. The system parameters used are listed below (see caption of Fig. 4). **a** Comparison between adiabatic (blue lines) and isothermal (orange lines) processes. Dotted lines represent ideal gas. **b** Examining the effect of *v*_atm_, the volume of the gas encapsulated in a single capsule at atmospheric pressure, and *k*_II_, the slope of phase II. The blue line is a reference curve based on experimental values, with *v*_atm_ = 18 ml and *k*_II_ = 152 kPa ml^−1^. The green and purple lines are pressure-density curves for fluid with decreased and increased values of *v*_atm_ of 12 ml and 24 ml, respectively. The red line is a pressure-density curve in which one of the unstable permutations is stabilized (see inset), achieved by setting *k*_II_ = 2300 kPa ml^−1^, while keeping all other parameters identical to the blue reference curve. The insets show that decreasing *v*_atm_ or increasing *k*_II_ creates regions where the fluid is stable although a bi-stable element is at the spinodal region.

The pressure-density relations of the fluid are presented in Fig. 3, where unstable states are marked by dashed lines and stable states are marked by solid lines. In Fig. 3a we present the external pressure *p* vs density *ρ* for adiabatic (blue lines) and isothermal (orange lines) processes. For a metafluid, the adiabatic process is associated with rapid actuation due to a limited heat transfer from the encapsulated gas to the surrounding liquid. For longer time periods, the gas temperature, surrounding liquid temperature, as well as the outside environment will balance, with an isothermal limit being appropriate.

In addition, pressure-density relations for gas only are presented by dotted lines with corresponding colors. The distance between stable points of the fluid is minimal near the permutation $\mathop{{{{{{{{\bf{per}}}}}}}}}\limits^{-\!\longrightarrow}=(0,0,n)$ and increases as we approach the reversed order permutation $\mathop{{{{{{{{\bf{per}}}}}}}}}\limits^{-\!\longrightarrow}=(n,0,0)$. In Fig. 3a, we show adiabatic processes, which were obtained for three different initial gas volumes within the capsules at atmospheric conditions (blue line is the reference curve, purple line has a decreased amount of gas, and the green line have an increased amount of gas). The effect of the amount of gas becomes more pronounced as the density increases and the average slope of the multi-stable curve increases with the addition of gas into the capsules. In addition, it is possible to see that permutations of the form $\mathop{{{{{{{{\bf{per}}}}}}}}}\limits^{-\!\longrightarrow}=({n}_{{{{{{{\mathrm{I}}}}}}}},1,{n}_{{{{{{{\mathrm{II}}}}}}}})$ become stable as the density of the fluid increases (see insets within Fig. 3b). The red curve in Fig. 3b, presents modified *k*_II_. In this case, a permutation with unstable element (*n*_s_ = 1) results in a stable state of the system. The insets show that decreasing *v*_atm_ or increasing *k*_II_ creates regions where the fluid is stable although a bi-stable element is at the spinodal region. In both panels, several stable densities are possible for a given pressure, as well as several pressures for a single density. This feature appears for unstable curves with positive slopes.

### Pressure cycles and experimental demonstration

**Fig. 4:**
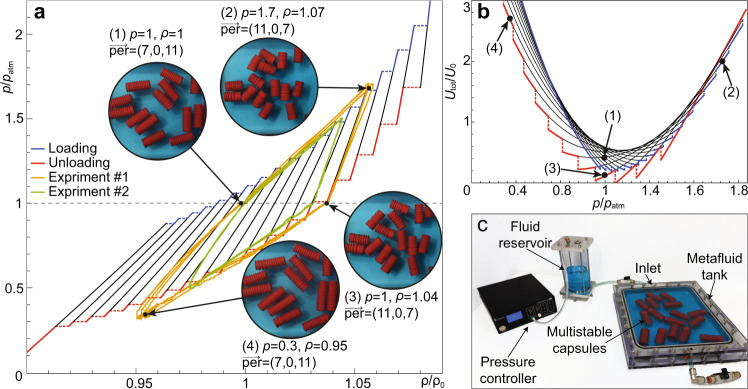
Pressure cycles and experimental demonstration. **a** Experimental and theoretical pressure (*p*/*p*_atm_) vs. density (*ρ*/*ρ*_0_) cycles. The black, blue, and red curves represent (analytically calculated) equi-permutation lines, loading, and unloading modes, respectively. Orange and green curves represent the experimental results (each curve containing three cycles). Representative frames (circular insets) taken from our experiment correspond to points (1)–(4) on the orange curve. In the experimental data, snap-open and snap-close points are (${v}_{{{{{{\mathrm{s}}}}}}}^{{{\mbox{I}}}},{p}_{{{{{{\mathrm{s}}}}}}}^{{{\mbox{open}}}}$) = (0.26 ml, 26.24 kPa) and (${v}_{{{{{{\mathrm{s}}}}}}}^{{{\mbox{II}}}},{p}_{{{{{{\mathrm{s}}}}}}}^{{{\mbox{close}}}}$) = (1.04 ml, −16.61 kPa), respectively. The slopes of the tri-linear approximation are *k*_I_ = 2300 kPa ml^−1^, *k*_II_ = 152 kPa ml^−1^, for phase I and II, respectively, and the number of bi-stable elements per capsule is 18. **b** The corresponding internal energy of the system, where points (1–4) correspond to the frames in **a**. **c** Experimental setup: the pressure controller, which is responsible for regulating the (constant) pressure in the fluid reservoir, is connected to the metafluid tank inlet via a flow valve. The height of the fluid in the fluid reservoir is monitored in the experiment to allow measuring the change in equivalent density of the metafluid. See Supplementary Movies 1–2.

In Fig. 4 we present analytic and experimental results for cyclical pressurization of the fluid. In Fig. 4a, each of the black curves denotes a stable equilibrium state corresponding to a different permutation, while the blue and red curves follow the actual loading and unloading paths, respectively, involving switching between different permutations. In order to access one of the multiple possible densities per a given pressure, we need to reach the specific permutation on which the desired density-pressure point is located. This can be achieved by increasing the pressure beyond the initial snap-close value associated with the current permutation (blue line in Fig. 4a), until reaching a stable permutation for the given pressure value. At this point, reducing the pressure will not change the permutation as long as the pressure is above the snap-open value of the current permutation (red line in Fig. 4a) and the system will remain at the same permutation (following the black line in Fig. 4a associated with that permutation). Thus, multi-stable pressure cycles are closed curves which involve intervals on constant permutation curves (black lines), intervals on the snap-close curves (blue lines), and intervals on the snap-open curves (red lines). This unique ability to choose a specific operation curve is demonstrated experimentally in what follows.

The experimental setup (Fig. 4c) consists of a cuboid 35 × 30 × 3.2 cm fluid-filled tank, a pressure flow controller, and a fluid reservoir. The tank contains 16 capsules, and is connected to a fluidic reservoir in which the pressure is regulated using a pressure controller (Elveflow OB1). Using this setup, we experimentally measured the parameters values of the a single capsule by eliminating the encapsulated gas effects (for more details, see Supplementary Notes 1). The following physical parameters were obtained: ${v}_{{{{{{\mathrm{s}}}}}}}^{{{{{{{\mathrm{I}}}}}}}}=0.26$ ml, ${v}_{{{{{{\mathrm{s}}}}}}}^{{{{{{{\mathrm{II}}}}}}}}=1.04$ ml, *k*_I_ = 2300 kPa ml^−1^, *k*_II_ = 152 kPa ml^−1^ and each capsule is composed of *n* = 18 bi-stable elements. In addition, we experimentally measured the snapping pressures (see definitions in Fig. 1) as ${p}_{{{{{{\mathrm{s}}}}}}}^{{{{{{{\mathrm{open}}}}}}}}=26.24$ kPa for the snap-open value and ${p}_{{{{{{\mathrm{s}}}}}}}^{{{{{{{\mathrm{close}}}}}}}}=-16.61$ kPa for the snap-close value, with standard deviation of ±0.1 kPa for both values. The measurements were based on averaging six experiments, where we used air actuation to eliminate transient fluidic effects. The surrounding fluid is water (*ρ*_*l*_ = 0.997 g ml^−1^) and the internal fluid is air (*γ* =1.4, *R* = 0.286 J g ^−1^ K^−1^). To obtain the change in density we measured the height of the fluid in the reservoir, which is equivalent to the change in the volume of the capsules (since the surrounding fluid is assumed incompressible). Throughout the experiment, the height of the liquid in the fluid reservoir and the pressure in the tank were continuously monitored. By using the density of the surrounding fluid and the volume of the metafluid tank, we calculated the equivalent density as a function of the pressure. The increase of tank volume due to elasticity of the walls was accounted for by pressurizing the tank without capsules, and measuring the volume-pressure relation.

In Fig. 4a, the orange and green curves present experimental results of two different pressure-density cycles of a multi-stable fluid. Each experimental curve presents three identical pressure cycles, repeated in each experiment. In Experiment #1, the initial state of the capsules at atmospheric conditions yields equivalent density of *ρ*_0_ = 0.719 g ml^−1^ and permutation of ${\mathop{{{{{{{{\bf{per}}}}}}}}}\limits^{-\!\longrightarrow}}_{0}=\{5,0,13\}$ (see Fig. 4a1). We pressurized the tank linearly from *p*_0_ = 101 kPa to 171 kPa over a period of 30 s. The fluid density increased and the permutations changed until reaching a steady state permutation of $\mathop{{{{{{{{\bf{per}}}}}}}}}\limits^{-\!\longrightarrow}=\{14,0,4\}$ and density of *ρ* = 1.07*ρ*_0_ (see Fig. 4a2). At this stage, we reduced the pressure back to *p*_0_ over a period of 30 seconds. In agreement with the model, the permutation of the fluid remained nearly unchanged ($\mathop{{{{{{{{\bf{per}}}}}}}}}\limits^{-\!\longrightarrow}=\{13,0,5\}$) while the density decreased to *ρ* = 1.037*ρ*_0_ (see Fig. 4a3). To return to the initial state, we applied a pressure of 30 kPa, which allowed us to change the state back to the initial fluid permutation of $\mathop{{{{{{{{\bf{per}}}}}}}}}\limits^{-\!\longrightarrow}=\{5,0,13\}$ and the density of *ρ* = 0.95*ρ*_0_ (see Fig. 4a4). Finally, increasing the pressure back to 101 kPa compressed the fluid along a constant permutation. Similarly, in Experiment #2 we applied a smaller cycle, in which points 1–4 have the densities of *ρ* = *ρ*_0_, *ρ* = 1.04*ρ*_0_, *ρ* = 0.97*ρ*_0_, *ρ* = 1.03*ρ*_0_ and the permutations of {7, 0, 11}, {11, 0, 7}, {11, 0, 7}, {7, 0, 11}, respectively. As it is evident from Fig. 4, a good agreement is observed between the theoretic stability states of the system and the experimental results for both cycles (see Supplementary Movies 1–2).

In Fig. 4b we present the internal energy associated with the different equilibrium states. The internal energy stored within the fluid at atmospheric conditions in the initial state, point 1, is released by adding sufficient energy to the fluid reaching a different permutation, point 2. From point 2, energy is released along a single permutation until reaching point 3, which is the minimal energy at the atmospheric conditions. Similarly, storing energy within the fluid can be achieved by temporarily adding energy to change permutations, and reaching point 4. Reducing the pressure back to atmospheric pressure will not change the fluid permutation, and some of the energy will thus remain stored within the fluid.

### Refrigeration cycles

Transfer of a metafluid between different permutations is analogous to phase-change in standard fluids. When the proposed metafluid snaps from one permutation to another, the internal gas rapidly and significantly expands or contracts with constant pressure. When a capsule is snapped open, the encapsulated gas cools down, and this translates to snap in heat absorption of the capsule, which is equivalent to the latent heat associated with evaporation. Similarly, snap-close is equivalent to condensation. In refrigeration cycles, phase-change is essential to achieving efficient cooling cycles^37,38^. As mentioned in the introduction, this often necessitates the use of environmentally hazardous gases with appropriate thermodynamic properties^39,40^. In this section, we discuss and characterize the operation of refrigeration cycles which utilize metafluids, with the aim of enabling the use of standard safe gases such as air for effective refrigeration without requiring phase change.

Cooling cycles in which the fluid remains in a gaseous state, such as the Brayton cycle, can operate with a variety of fluids, with air being the most commonly used. Such cycles, known as air cycle refrigeration (ACR), were for many decades the predominant technology for air conditioning of jet engine-driven airplanes, due to their relatively simple working mechanism and compactness. However, their performance is quite limited in comparison to vapor-compression cycles^41^, in which the refrigerant undergoes phase change throughout the cycle presenting enhanced performance thanks to the ability to absorb additional heat in the form of latent heat.

**Fig. 5:**
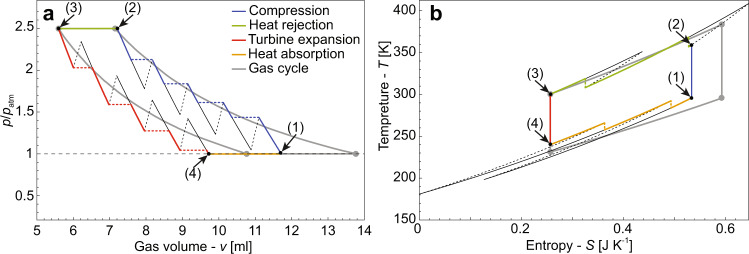
Refrigeration cycles simulations of a metafluid compared with gas only. **a** Pressure volume diagram of a metafluid cycle, transition between states 1-2, 2-3, 3-4, and 4-1 denoted by black green, red, and orange lines, respectively, and air cycle denoted by gray lines. **b** Temperature-Entropy diagram.

The thermodynamic cycle of a standard, gas only, reversed Brayton refrigerator^42^ is presented in Fig. 5 (solid gray lines). This cycle is used to remove heat from a conditioned reservoir, state 1, where the temperature is *T*_1_ = *T*_c_ and the pressure is *P*_1_ = *P*_atm_. The heat is transferred to the surrounding reservoir, state 3, where the temperature is *T*_3_ = *T*_room_ and the pressure is *P*_3_ = *αP*_1_ (*α* > 1 is the compression ratio). Let us assume that the gas enters the compressor at state 1. The gas is then compressed to state 2, where the pressure is *P*_2_ = *αP*_1_, in an adiabatic and reversible process which causes the gas to heat up, reaching *T*_2_, which is well above the surrounding temperature, *T*_2_ > *T*_3_. During this process, the pressure-volume relation follows from the equation $P={P}_{1}{\left({V}_{1}/V\right)}^{\gamma }$ and the temperature satisfies $T={T}_{1}{(V/{V}_{1})}^{1-\gamma }$. After stage 2 is reached, the refrigerant comes in contact with the surrounding reservoir and is cooled down to state 3 in an isobaric process. Next, the gas enters a turbine, which causes the gas to expand back to *P*_4_ = *P*_1_, reaching the temperature of *T*_4_ which is below that of the conditioned region. After stage 4 is reached, the refrigerant comes in contact with the conditioned reservoir and is heated up to state 1 in an isobaric process.

The diagrams of a metafluid cooling cycle are presented in Fig. 5, along with those of the standard Brayton cycle using air. In Fig. 5a, we show a pressure volume diagram and in Fig. 5b we show a temperature-entropy diagram. The blue, green, red, and orange lines in Fig. 5 correspond to metafluid transitions 1-2, 2-3, 3-4, and 4-1, respectively. Gray lines denote standard air based Brayton cycle. The properties of the metafluid are the same as those presented in the previous section, and are based upon experimentally measured values. The externally applied pressure is different from the pressure of the gas, and isentropic relations yield ${p}_{{{{{{\mathrm{ex}}}}}}}={P}_{1}{\left({V}_{1}/V\right)}^{\gamma }-{p}_{{{{{{\mathrm{el}}}}}}}$, where *p*_el_ depends on the permutation of the metafluid, $\mathop{{{{{{{{\bf{per}}}}}}}}}\limits^{-\!\longrightarrow}=({n}_{{{{{{{\mathrm{I}}}}}}}},{n}_{{{{{{{\mathrm{s}}}}}}}},{n}_{{{{{{{\mathrm{II}}}}}}}})$, and is calculated via Eq. (2).

During the entire cycle, multiple snaps of the metafluid occur between different permutations. In order to quantify the efficiency of a metafluid-based cooling cycle, the coefficient of performance (COP) is calculated. The COP is defined as the ratio of the refrigeration effect to the net work input. Net work is calculated as the work performed on the compressor less the work performed by the turbine, and refrigeration effect is measured as heat transferred between states 4 and 1. The COP is given by

13 $${{{{{\mathrm{COP}}}}}}=\frac{{\dot{Q}}_{{{{{{\mathrm{C}}}}}}}}{{\dot{W}}_{{{{{{\mathrm{in}}}}}},{{{{{\mathrm{net}}}}}}}}.$$

See Supplementary Notes 2 for a detailed description of the calculation.

Based on the exact parameters used in the experiment (see Fig. 4), we found that the metafluid demonstrated COP of 2.29, which is significantly higher than the Brayton air based cooling cycle’s COP of 1.4. The efficiency improvement of 61% was achieved without any optimization of the system. In Supplementary Notes 3 we present the effect of various parameters, such as the number of bi-stable elements in the capsule and the mass of the encapsulated gas. In the figures presented in the Supplementary Notes, all of the system’s parameters except for one are held constant. The results show that the efficiency of the system (COP) is mainly determined by the number of snaps occurring during the heat exchanging segments of the refrigeration cycle. Further optimization can be achieved if we modify the properties of the bi-stable frusta, showing optimal value of about $0.3{k}_{{{{{{\mathrm{I}}}}}}}^{{{{{{\mathrm{exp}}}}}}}$ in which there is COP value of 8.28, demonstrating that metafluids have the potential to replace vapor compression cycles and enable safe and efficient refrigeration while using common and safe gases, such as air.

## Discussion

In this study, we introduce a new concept for a multistable metafluid, and investigate theoretically and experimentally the relationship between pressure and density both for adiabatic and isothermal processes. As a result of the variety of stable densities and energy states, it is possible to capture and store energy at standard atmospheric conditions, which is not possible with naturally occurring fluids. Due to these properties, metafluids are relevant to the ubiquitous refrigeration and heat cycles, as well as many other applications such as the creation of flow fields with physical memory attributed, as demonstrated in recent studies in the context of metamaterials^43,44^.

The experiments presented in this work involved large *O*(cm) particles. While these experiments are aimed at demonstrating the concept, future research on miniaturization of the multistable particles is needed for creating metafluids for real applications. Minimization of the capsules may be achieved by utilizing a wide range of established manufacturing technologies^45^, such as lithography^46,47^, high-resolution additive manufacturing^48,49^, and propagating photo-polymer wave-guide prototyping^50^, which were proven to enable bi-stable or multistable elements.

We examined the application of such a metafluid to refrigeration cycles and presented significant improvements to the efficiency of such cycles. We note however, that in order to realize a refrigeration device based on a metafluid, various other requirements need to be taken into account, such as optimizing heat transfer rates, as well as creating a system that does not damage the capsules. These considerations may alter the COP values for an actual device, and will need to be addressed in a future research. The results of this work suggest that the concept of multistable metafluids paves the way for the creation of futuristic non-polluting fluids with enhanced properties for energy and cooling cycles, leveraging multi-stability to harvest, store and release energy and heat.

## Methods

Details on the geometry, design, fabrication, testing, analytical model, and numerical solutions are provided in the Supplementary Information.

## Supplementary information

Supplementary Information

Description of Additional Supplementary Files

Supplementary Movie 1

Supplementary Movie 2

Supplementary Movie 3

Supplementary Software 1

## Data Availability

The thermodynamic analysis of the presented metafluid (Figs. 2–5 and the Supplementary Information) generated in this study have been deposited in the Figshare database under accession code 10.6084/m9.figshare.14988048^51^.

## Code availability

All codes used for simulations (Mathematica) and experimental results processing are publicly accessible at 10.6084/m9.figshare.14988048^51^.
