# Supplementary material for: A metafluid with multistable density and internal energy states
Source: Nat Commun. 2022 Apr 5;13:1810. doi: 10.1038/s41467-022-29048-3 (PMC8983681; doi:10.1038/s41467-022-29048-3)
Supplement: Supplementary file 1 — Supplementary Information [file 41467_2022_29048_MOESM1_ESM.pdf]

# Supplementary Information for
## "A metafluid with multistable density and internal energy states"

**Ofek Peretz[1, *], Ezra Ben Abu[1], Anna Zigelman[1], Sefi Givli[1], and Amir D. Gat[1]**
[1]Faculty of Mechanical Engineering, Technion - Israel Institute of Technology, Haifa 3200003, Israel.

[1]To whom correspondence should be addressed.*ofekperetz@campus.technion.ac.il

### Supplementary Information
**Supplementary Note 1: Fabrication**

To fabricate the multistable capsules, we used a commercially available (Poof-Slinky Inc., USA) structure which is made of poly (vinyl siloxane) elastomer with an equivalent Elastic modulus of $E = 1.33$ MPa. The bistable element dimensions are: inner radius $r_i = 11.1$ mm, outer radius $r_0 = 14.65$ mm, thickness $t = 0.5$ mm. The lengths of an element are: in the undeformed open state $h_{open} = 5.8$ mm and in closed state $h_{close} = 1.45$ mm. To measure the snapping pressures, $(p_s^{open}, p_s^{close})$, and the associated volume values, $(v_s^I, v_s^{II})$, we examined a single capsule in a pressure tank (the inner gas effects were eliminated by exposing the inner volume of the capsule to the atmospheric conditions via a connecting tube). The volume of the capsule was then measured for different values of the external pressure. **Supplementary Figure 1** shows experimental results (three experiments marked by different colors) for pressure versus volume of a single capsule. As the effects of the internal gas were eliminated, all bistable elements are expected to snap for the same pressure, according to which we calculated the values of the snapping pressures (see definitions in Fig. 1 in the main text). The snapping pressures are $p_s^{open} = 26.24$ kPa for the snap-open value and $p_s^{close} = -16.61$ kPa for the snap-close value, with standard deviation of $\pm 0.1$ kPa for both values. Furthermore, the point where the structure changes its permutation defined the stability threshold point which satisfies $(n \cdot v_s^I, n \cdot v_s^{II})$. The experimental results yielded $n \cdot v_s^I = 4.68$ ml and $n \cdot v_s^{II} = 18.72$ ml, thus, since $n = 18$, resulting in $v_s^I = 0.26$ ml and $v_s^{II} = 1.04$ ml. To estimate the values of $k_I, k_{II}$, we measured the slope of the pressure volume relation at known permutation regions. For example, at pressure values higher then $p_s^{close}$, the permutation of the capsule is $\overrightarrow{\boldsymbol{per}} = \{18, 0, 0\}$ and therefore the slope in the experiment corresponds to $n = 18$ identical springs with stiffness $k_I$ connected in series, which yields $k_I = k_I^{exp}/n = 2300$ kPa ml$^{-1}$. In the same manner we calculated $k_{II} = k_{II}^{exp}/n = 152$ kPa ml$^{-1}$. The stiffness value at the spinodal region was estimated according to the tri-linear assumption via $k_s = (p_s^{close} - p_s^{close})/(v_s^{II} - v_s^I) = 54.9$ kPa ml$^{-1}$.

***Supplementary Note 2: Detailed analysis of refrigeration cycle***

In Section 6 in the main text, we present simulations of a reversed Brayton cycle using our metafluid as a refrigerant and compare our results with that of a standard gas refrigerant. Below, a more detailed analysis of the derivation used to generate Fig. 5 (in the main text) is provided. To simulate a refrigeration cycle, we first need to define the relations between entropy, pressure, and temperature of the refrigerant. The working fluid is air, assumed to be an ideal, calorically perfect gas (having a constant specific heat). All processes within the closed-cycle are assumed reversible. Under the above assumptions, the second law of thermodynamics takes the form,

$$(ds)_{ideal\ gas} = c_p \left(\frac{dT}{T}\right) - \frac{v}{T} dp, \tag{1}$$

where $s$ is the entropy, $p$ is the pressure, $T$ is the temperature, $v$ is the volume, and $c_p$ is the specific heat capacity, which we assume to be constant in our analysis. Integrating the above equation between point 1 to point 2, we get

$$\Delta s = c_p \ln\left(\frac{T_2}{T_1}\right) + R \ln\left(\frac{v_2}{v_1}\right), \tag{2}$$

where $R$ is the universal gas constant, $T_1$, $T_2$ are the temperatures at states 1 and 2, respectively, and $v_1$, $v_2$ are the corresponding volumes. Typically, Brayton cycle consists of two types of processes: Isentropic and Isobaric. Let us

simplify the equations in both cases. In isentropic process, which means adiabatic and irreversible compression, there is no change in entropy, so equation (1) yields that,

$$\frac{T_2}{T_1} = \left(\frac{v_2}{v_1}\right)^{1-\gamma} = \left(\frac{p_2}{p_1}\right)^{\frac{\gamma-1}{\gamma}} \tag{3}$$

Thus, using the ideal gas law, we get that $p_1 v_1^{\gamma} = p_2 v_2^{\gamma}$, where $\gamma$ is the adiabatic index (for air we use $\gamma = 1.4$). In the isobaric process, the pressure is kept fixed, namely $p = p_1 = cosnt.$, which substituted into equation (1) yields that,

$$\Delta s_{1,2} = c_p \ln\left(\frac{T_2}{T_1}\right), \qquad \frac{T}{V} = \frac{T_2}{V_2} \tag{4}$$

**Calculation of Brayton cycle states.** We consider the case of a reversible cycle, presented by points 1-2s-3-4s. This cycle is used to remove heat from a conditioned reservoir (denoted by state 1), where the temperature is $T_1 = T_c$ and the pressure is $p_1 = p_{atm}$. The ambient reservoir, (denoted by state 3) whose temperature and pressure are denoted by $T_3 = T_h$ and $p_3$, respectively, is hotter than the conditioned reservoir ($T_3 > T_1$) and therefore direct, spontaneous, heat transfer cannot be used. To reach state 3 from state 1, the gas is first compressed from state 1 to state 2 in an isentropic process, where the temperature $T_{2s}$ can be calculated by using equation (3), namely

$$T_{2s} = T_1 \left(\frac{p_{2s}}{p_1}\right)^{\frac{\gamma-1}{\gamma}} = T_1 \alpha^{\frac{\gamma-1}{\gamma}} \tag{5}$$

Next, the working fluid transfers heat to the ambient reservoir at a constant pressure, so that its temperature decreases from $T_{2s}$ to $T_3$. Afterwards, the pressure is released, which causes the gas to expand from state 3 to state 4 in an isentropic process and the temperature $T_{4s}$ can be calculated by using equation (3), namely

$$T_{4s} = T_3 \left(\frac{p_{4s}}{p_3}\right)^{\frac{\gamma-1}{\gamma}} = T_3 \alpha^{\frac{1-\gamma}{\gamma}} \tag{6}$$

Lastly, the working fluid absorbs heat from the conditioned reservoir at a constant pressure, so that its temperature increases from $T_{4s}$ to $T_1$. To summarize, all states of the cycles are well defined as a function of the parameters $p_{atm}, T_c, T_h$ and $\alpha$ for a gas only cycle.

**Solution of Brayton cycle in a metafluid.** When simulating a metafluid as a refrigerant in the above cycle, some modifications should be taken into account. First, the cycle is pressure controlled, and in terms of our metafluid, it means that we monitor the external pressure. This pressure (denoted by *p*) is different from the pressure of the encapsulated gas (denoted by $p_{gas}$) and the difference between them equals to $p_{el} = p_{gas} - p$ (see Section 2, paragraph 2 in the paper). Secondly, the volume of the metafluid is a multi-valued function of the difference between the pressure of the internal gas and is history dependent. Therefore, to simulate such a cycle, we start from a specific initial permutation and monitor the snaps in each process according to a pressure-controlled cycle (presented in Section 5 in the paper). After the first cycle, in which the initial condition was chosen arbitrarily, the next simulation is cyclic (starts and ends in the same state).

**Coefficient of performance.** The efficiency of such cycle is measured by the coefficient of performance (COP), which is the ratio between the amount of heat rejected from the conditioned reservoir, which we denote by $\dot{Q}_c$, and the net work done on the system, which we denote by $\dot{W}_{in,net}$. The first term, $\dot{Q}_c$, is given by

$$\dot{Q}_c = c_p(T_1 - T_4) > 0 \tag{7}$$

and the net work, $\dot{W}_{in,net}$, is calculated by the amount of work done by the compressor ($\dot{W}_c$) minus the work done by the turbine ($\dot{W}_t$). These are calculated by,

$$\dot{W}_c = c_p(T_2 - T_1), \qquad \dot{W}_t = c_p(T_3 - T_4) \tag{8}$$

Note that so far, we assumed an ideal cycle, but in reality, both the compressor and the turbine have non-ideal efficiencies. Thus, we denote by $\eta_c$ the efficiency of the compressor and by $\eta_t$ as the efficiency of the turbine. Using the above relations, we can calculate the COP by,

$$COP = \frac{\dot{Q}_c}{\dot{W}_{in,net}} = \frac{T_1 - T_4}{(T_{2s} - T_1)/\eta_c - (T_3 - T_{4s})\eta_t}. \quad (9)$$

Where $T_4 = T_3 - (T_3 - T_{4s})\eta_t$. In our simulations which follow, we assumed that $\eta_t = \eta_c = 85\%$, which is typical to such system (see reference [42] in the paper).

***Supplementary Note 3: Design rules for metafluids in refrigeration cycles***

This section presents some design rules that can be applied to optimize the performance of a metafluid for a particular application or goal. While the proposed metafluid can be applied in a wide variety of applications, we will focus in our discussion on a refrigeration cycle (as discussed in Section 6 of the paper). The efficiency of such cycle, denoted by COP, is determined by the ratio between the amount of heat transferred from the conditioned region to net work done on the system. In Supplementary Figure 2, we simulated a refrigeration cycle using our metafluid and compared it to the pure gas cycle. In all simulations, we kept all the parameters fixed (with values similar to the ones used in our experiment) and changed only the amount of gas within the capsules (measured by the volume taken by the gas contained within a single capsule at atmospheric pressure $v_{atm}$ in the paper) each simulation, we calculated the COP ratio (panel a) and the number of snaps between permutations (panels b, c) versus $v_{atm}$ in each process. In panel b, we show the number of snaps in the heat exchanging processes (the number of snaps in process (2-3) is denoted by green line and in process (4-1) it is denoted by orange line). In panel c, we show the number of snaps in the compression/expansion processes (the number of snaps in process (1-2) is denoted by blue line and in process (3-4) it is denoted by red line). The results indicate that the maximal efficiency (denoted by green-shaded regions) is achieved for cycles that involve a combination of maximal number of snaps in process (2-3) and minimal number of snaps in process (4-1). Overall, our results suggest that elastic multistability of metafluids (permutation "phase" change) and standard phase change are analogous in the sense that latent heat in metafluids can be defined as the net heat emitted or absorbed during a transition between different permutations.

Using the same geometrical properties of capsules used in the experiment, and choosing the optimal value for the mass of the encapsulated gas, we obtain COP value of 2.29 (improvement of 61% compared to gas only cycle) as presented in Supplementary Figure 3.

In Supplementary Figure 3 we repeated the simulations described above for different values of $k_I$ (all other values are based on the experimental configuration). The results indicates that an improvement of up to $500\%$ in COP relative to standard gas cycle can be achieved by optimizing the values of $k_I$ and the amount of gas in the capsules $v_{atm}$.

Another key parameter in the design of such capsules is the number of bistable elements in each capsule. To give a guiding line to the choice of this number, we plotted COP relative to gas only cycle as a function of the number of bi-stable elements in each capsule. In each simulation, we kept the overall volume of the capsule constant (and all other parameters were kept fixed) and changed the number of capsules from $n = 1$ to $n = 50$. Supplementary Figure 3 shows the relative COP versus the number of bi-stable elements ($n$). It can be seen that in order to overcome the reference cycle, in terms of higher COP, a minimal number of bistable elements satisfies $n > 8$. Note, that even for a large number of bi-stable elements in a capsule, some cycles show significant decrease in performance. This non-homogeneous behavior of COP versus $n$ may be explained by a non-homogeneous behavior of the number of snaps. In these regions, the COP may be increased by tuning some other parameters of the metafluid, which will allow to preserve the same (optimal) number of snaps.

The selection of the external fluid is an additional degree of freedom in the design of metafluids. It is the primary function of immersion of the capsules in a fluid is only to lubricate the capsules. Fluids intended for immersion should

be as stable as possible and have the lowest possible viscosity. It is also important to consider gravity when designing capsules and containers in which flow occurs. Depending on the configuration in which the capsules are arranged, the capsules either settle or float due to gravity. To minimize this effect, the mass of the capsules should be adjusted in order to achieve neutral buoyancy in the middle of the operating range.

***Supplementary Note 4: Effects of gravity***

The experiments presented in figure 4 in the main text were performed in a shallow rectangular box, with dimensions of 35 x 30 x 3.2 cm. In the presented experiments, gravity was directed in the narrow direction, and thus did not affect the distribution of capsules in the $x-y$ plane which was the focus of the experimental results. Furthermore, since the gravity induced pressure difference within the narrow direction scales as $\rho g h_z \approx 3 \times 10^2 Pa$, while the actuated pressures are $\approx 5 \times 10^4 Pa$, the effect of gravity on transition between permutations was negligible.

However, other configurations may have significant gravity effects. Even in situations where gravity does not induce sufficient pressure to transition between permutations, buoyancy effects may cause the capsules to sink, to float, or to suspend in a natural buoyancy location. This will be determined by the relative density $R_d$ between the capsules and the ambient fluid, which is given by the ratio

$$R_d = \frac{m_c V_c}{\rho_f}.$$

As a means of examining the impact of gravity on pressure-density cycles, we repeated the experiments presented in the main text (specifically Figure 4) with gravity oriented along the long $y-$axis. In this case, gravity-induced pressure will be scaled as $\rho g h_y \approx 3 \times 10^3 Pa$, and capsules will be translated along the $x-y$ plane during the cycle. The obtained results, as well as reference data without gravity, are presented in Supplementary Figure 5. Although the spatial distribution of the capsules did change over the pressure cycle, Supplementary Figure 5 shows no significant change in the pressure-density curve, which suggests that the averaged thermodynamic properties of the suspension are not significantly affected by gravity.

Buoyancy affects the meta-fluid behavior by changing the spatial distribution of the capsules within the fluid, and the transitions between permutations due to the hydrostatic pressure distribution created within the fluid. The effect of buoyancy on the spatial distribution cannot be fully neglected for all states, regardless of the size of the capsules. Creating a uniform distribution of particles can be achieved for shallow containers with gravity acting in the narrow axis, as presented in the experiments, or by creating repulsion forces between the particles (e.g. via electric forces). The effect of buoyancy on transitions between permutations due to the hydrostatic pressure distribution, is negligible when the hydrostatic pressure is much smaller than the snapping pressures, which expressed by the requirement $\rho g l \ll p_s^{open}$.

## Supplementary Figures

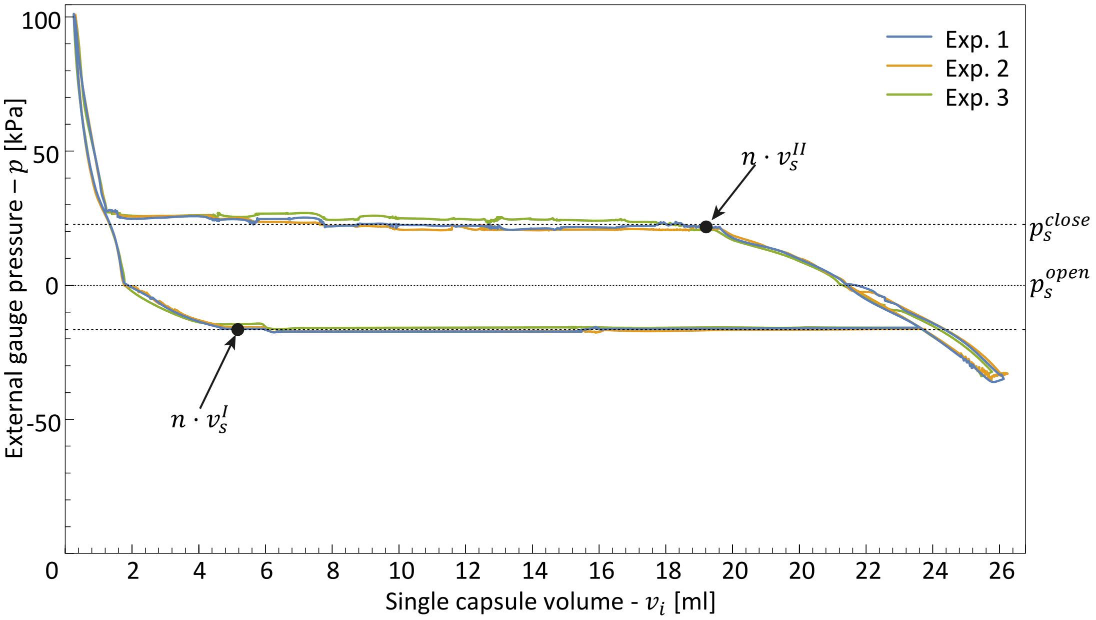


**Supplementary Figure 1- Experimental characterization of capsules properties.** Three experimental results of pressure versus volume diagram are presented for a single capsule without encapsulated gas effects. By eliminating the internal gas effects, we characterized the properties the elastic structure. Averaging the results of three experiments (each on a different capsule) we measured the following values: $v_s^I = 0.26$ ml, $v_s^{II} = 1.04$ ml, $k_I = 2300$ kPa ml$^{-1}$, $k_I = 152$ kPa ml$^{-1}$, $p_s^{open} = 26.24$ and, $p_s^{close} = -16.61$. These values were used in all simulations presented in Figs. 2-4 in the main text and shows good agreement with the experimental results.

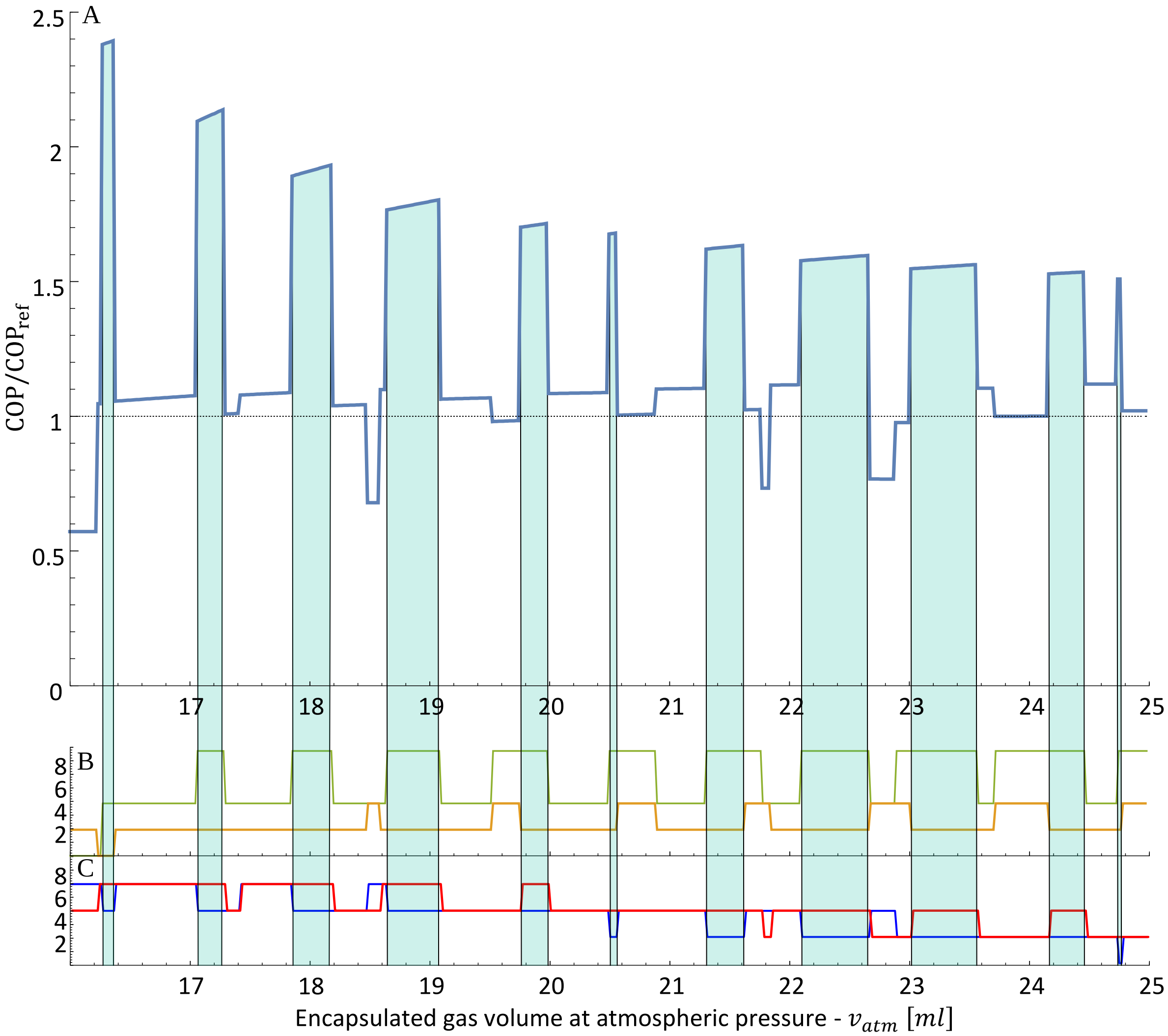


**Supplementary Figure 2 - Relative COP versus encapsulated gas volume.** Plotted values are normalized by those of a gas-only cycle. All parameters are identical to those of our experiment, with (only) the atmospheric volume of the encapsulate gas (equivalent to the amount of gas in each capsule) being varied in different simulations. **A** Relative COP simulation results versus the atmospheric volume of the encapsulated gas $\boldsymbol{v_{atm}}$. **B** Number of snaps in the heat exchanging processes (the number of snaps in process (2-3) is denoted by a green line and in process (4-1) is denoted by a orange line). **C** Number of snaps in the compression/expansion processes (the number of snaps in process (1-2) is denoted by a blue line and in process (3-4) is denoted by a red line).

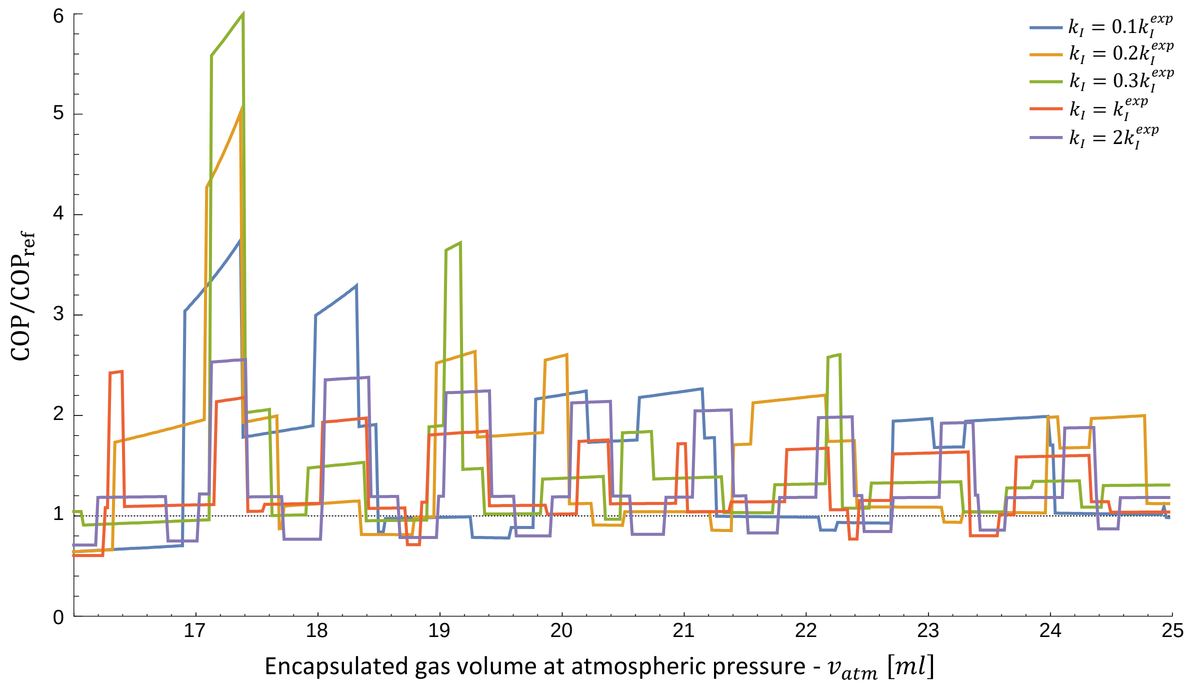


**Supplementary Figure 3 - Relative COP versus encapsulated gas volume for different values of $k_I$.** Plotted values are normalized by those of a gas-only cycle. All parameters are identical to those of our experiments, with (only) the atmospheric volume of the encapsulate gas (equivalent to the amount of gas in each capsule) being varied in different simulations. Each line presents relative COP vs encapsulated gas for different value of $k_I = \{0.1, 0.2, 0.3, 1, 2\} k_I^{exp}$, where $k_I^{exp} = 2300\ kPa\ ml^{-1}$. The results indicate that an improvement of up to 500% can be achieve for $k_I = 0.3k_I^{exp}$ and $v_{gas} = 17.46\ ml$.

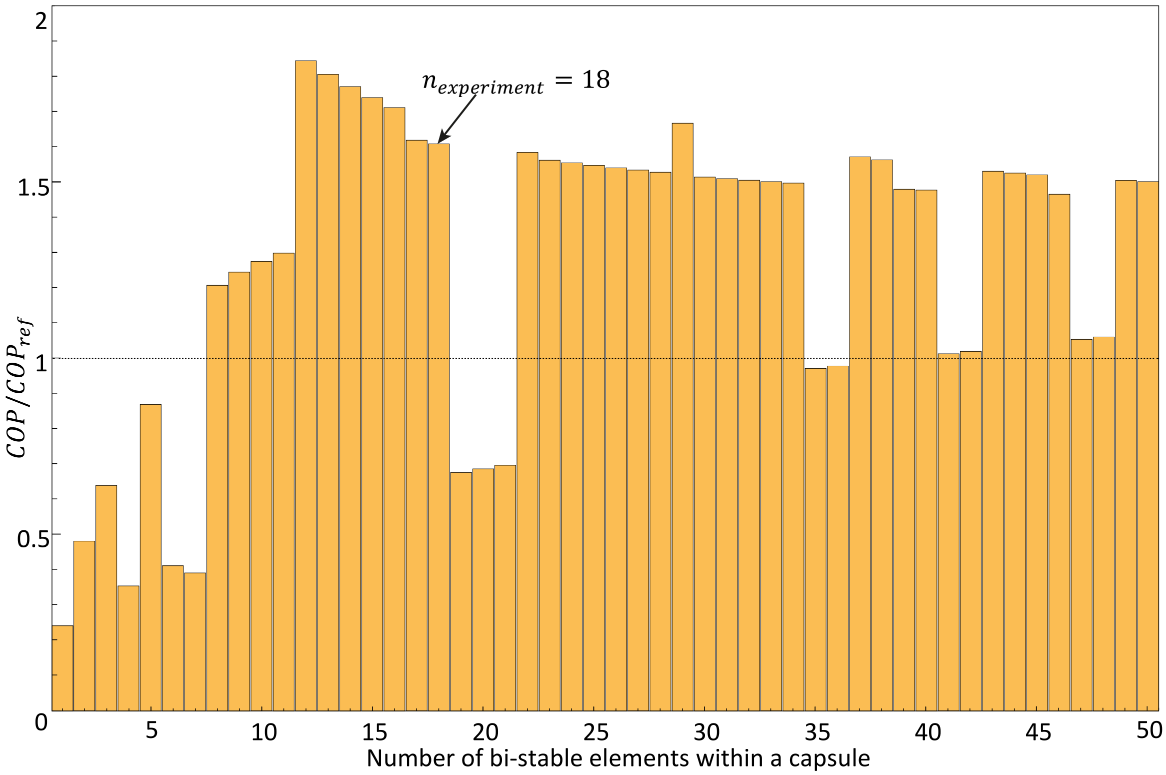


**Supplementary Figure 4 - Relative COP versus the number of bi-stable elements in each capsule.** In all simulations, we kept the overall volume of the capsule constant (and all other parameters were kept fixed) and changed the number of capsules from $n = 0$ to $n = 50$.

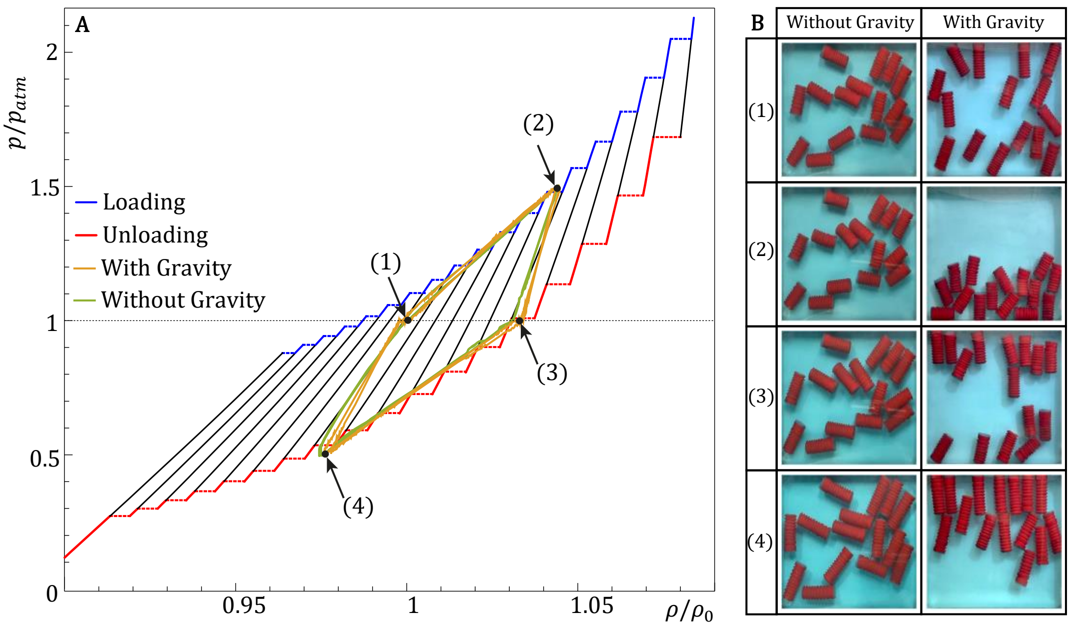


**Supplementary Figure 5 –** Pressure cycle comparison between system with gravity vs without gravity. **A** Experimental and theoretical pressure ($p/p_{atm}$) vs. density ($\rho/\rho_0$) cycles. The black, blue, and red curves represent (analytically calculated) equi-permutation lines, loading, and unloading modes, respectively. Green curve represents the experimental results of a system without the presence of gravity (identical to Figure 4 in the main text) and the orange represents the experimental results of a system with the presence of gravity (each curve containing two cycles). **B** showing Representative frames taken from the experiment correspond to points (1)–(4) comparing system behavior under gravitational effects. See Supplementary Movie #3.
