# Supplementary material for: A metafluid with multistable density and internal energy states
Source: Nat Commun. 2022 Apr 5;13:1810. doi: 10.1038/s41467-022-29048-3 (PMC8983681; doi:10.1038/s41467-022-29048-3)
Supplement: Supplementary file 2 — Description of Additional Supplementary Files [file 41467_2022_29048_MOESM2_ESM.pdf]

Description of Additional Supplementary Files

File Name: Supplementary Movie 1
Description: Experimental data of pressure and density used to generate Experiment #1 curve in Fig.4.

File Name: Supplementary Movie 2
Description: Experimental data of pressure and density used to generate Experiment #2 curve in Fig.4.

File Name: Supplementary Movie 3
Description: Experimental data of pressure and density with the presence of gravity used to generate used in Supplementary Figure 5.

File Name: Supplementary Software 1
Description: Simulation code (in Mathematica) used to calculate analytical curves in figures 2-4 and refrigeration cycle simulations presented in Fig. 5.
